# The effect of implementation leadership training for nursing informal leaders in the evidence-based practice

**DOI:** 10.3389/fmed.2025.1631810

**Published:** 2025-07-31

**Authors:** Xueyan Huang, Can Wang, Lumeng Lu, Jianao Chen, Rui Wang, Ying Feng, Yuhong Dong, Haifang Zhou

**Affiliations:** ^1^Department of Nursing, Hangzhou TCM Hospital Affiliated to Zhejiang Chinese Medical University, Hangzhou, China; ^2^Department of Orthopaedics, Hangzhou TCM Hospital Affiliated to Zhejiang Chinese Medical University, Hangzhou, China; ^3^Department of Science and Education Section, Hangzhou TCM Hospital Affiliated to Zhejiang Chinese Medical University, Hangzhou, China; ^4^Department of Massage, Hangzhou TCM Hospital Affiliated to Zhejiang Chinese Medical University, Hangzhou, China

**Keywords:** informal leaders, implementation leadership training, Kirkpatrick’s evaluation, evidence-based practice, Ottawa model

## Abstract

**Background:**

Implementation leadership is important for the successful implementation of evidence-based practice (EBP). Informal leaders, who are important promoters of EBP in nursing in the current healthcare system, can affect nursing management, organizational effectiveness, and cultural quality positively. However, informal leaders may lack training in leadership management and EBP. This study evaluated the effectiveness of the training program for implementation leadership, aiming to improve the leadership of informal leaders in EBP.

**Methods:**

Based on the Ottawa Model, this study designed a training program for implementation leadership, which lasted for 4 months and had 60 class hours. Seventy-five nursing informal leaders were trained in three steps of “Theoretical Training-Interactive Workshop-Theme Report”. Before the first training and after the last training, we evaluated the training effectiveness using Kirkpatrick’s evaluation model at the four levels of reaction (training satisfaction survey), learning (Evidence-Based Practice Belief Scale), behavior (Evidence-Based Nursing Competence Scale), and result (Implementation Leadership Scale). Data were analyzed by descriptive statistics, and paired t-tests for effect sizes.

**Results:**

At the reaction level, the informal leaders had 100% engagement and high satisfaction score. At the learning level, the score of the Evidence-Based Practice Belief Scale of informal leaders after training [(69.24 ± 5.32)] vs. [(59.91 ± 5.96)] was significantly higher than that before training (*p* < 0.001). At the behavior level, the score of the Evidence-Based Nursing Competence Scale of informal leaders after training [(74.47 ± 5.75) vs. [(56.37 ± 7.15)] was significantly higher than that before training (*p* < 0.001). At the result level, the score of the Implementation Leadership Scale of informal leaders after training [(38.88 ± 2.76) vs. [(30.01 ± 3.24)] was significantly higher than that before training (*p* < 0.001).

**Conclusion:**

The training program is a highly accepted, practical, and effective implementation strategy for informal leaders, which can improve the evidence-based nursing belief, evidence-based nursing competence, and implementation leadership of nursing informal leaders, and has a positive impact on EBP.

## Background

As the largest group of practitioners in the healthcare system, nurses have great potential in improving the health outcomes of patients and the overall medical quality through implementing the best evidence during the implementation of evidence-based practice (EBP) (1). According to the International Council of Nurses, nursing EBP is defined as “a method to solve clinical decision-making problems, including finding the best and latest evidence, clinical expertise and evaluation, and patient preference value in the context of nursing” (2). Recent studies have found that most nurses recognize EBP’s positive effects on patients, but their evidence-based practice ability is relatively low (3), and they lack confidence in evidence synthesis and transfer (4). In nursing practice, the implementation of evidence is a slow process (5). The EBP implementation is limited by many factors, including knowledge, cognition, convention, environmental background, cultural influence, leadership, patient preferences, organizational resources, etc. (6, 7). Therefore, narrowing the gap between evidence-based evidence and clinical practice, implementing EBP, and promoting organizational change and development have attracted much attention in the field of evidence-based nursing.

Implementation leadership refers to the multi-dimensional implementation process that can promote the use of evidence by clinical staff in clinical decision-making, including the impact of unit managers or supervisors on employees, the environment, and organizational infrastructure (8). Recently, it is confirmed that implementation leadership plays an extremely important role in promoting the successful implementation and maintenance of EBP (9). Specifically, implementing leadership behaviors improves the organizational EBP implementation climate and promotes the adoption of EBP by clinicians, leading to sustained practice change (9). However, empirical analyses confirm that the effectiveness of EBP implementation is complex and depends on the coordination between leaders at all levels (10). The multi-level leadership model believes that the leading methods of senior and middle managers can provide information for evidence-based implementation strategies and affect the attitude of front-line employees in providing evidence-based medical care practices. The first-level leaders, who directly supervise and manage front-line employees (11), have unique advantages in supporting senior leaders to implement healthy behavior EBP (12). In addition to the formal leaders of different levels, the institutionalization of EBP and organizational change requires the proactive and meaningful participation of informal leaders (13). It is increasingly recognized that the subjects of implementation leadership not only include the people who are endowed with formal power in the organization but also the informal leaders who do not hold positions but still execute leadership functions and exert influence on colleagues (14, 15).

Within China’s tiered healthcare system, managerial receptiveness to evidence adoption may facilitate successful implementation (16). Nursing informal leaders, typically exemplified by senior clinical nurses and specialty nurses not holding administrative titles, serve as critical bridges between policy directives and frontline practice. Unlike nurse educators in North America who typically occupy formal leadership roles, Chinese informal leaders demonstrate less workplace empowerment and lack decision-making autonomy due to their absence of formal administrative titles (17). Their influence derives from clinical seniority, collectivist cultural norms, and peer recognition rather than formal authority. Crucially, they drive EBP implementation through: (1) direct application of evidence at the point-of-care, (2) influencing team behaviors via informal channels (e.g., shift handovers, social media groups), and (3) relaying implementation barriers to formal leaders for strategy adjustment. They can affect the performance, efficiency, and culture of organizations in a positive way (18). Cranley et al. identified nine roles that could promote EBP, including opinion leaders, teachers, winners, practice promoters, etc. (19). All of these roles are assumed by nursing informal leaders, who are strategic thinkers, constantly assimilating and analyzing information and helping the team to make better decisions (18), creating a link between nursing wards and formal leaders, and motivating team members to achieve common goals (20).

Empirical studies have shown that informal leaders play a positive role in implementing evidence, providing practical solutions to patient safety problems, and improving patient satisfaction (21, 22). However, informal leaders in China lack formal leadership training; their implementation leadership is insufficient; and they need further training to effectively implement EBP-related leadership behaviors (1, 23). Cassidy et al. found that multi-component educational intervention and participation strategies were present in multiple stages of evidence-based implementation (1). Therefore, informal leaders must receive well-designed training programs to improve their ability and decision-making and management skills for effective implementation of practice leadership (24), better promoting evidence implementation.

Leadership training programs have been increasingly developed. For example, Aarons et al. developed the leadership and organizational change for implementation (LOCI) (25) to improve the implementation environment (26) and strengthen the implementation leadership of first-level leaders through multifaceted and multi-level implementation strategies. Procter et al. designed the training in implementation practice leadership (TRIPLE), and found that the implementation leadership behaviors and unit implementation climate of middle-level leaders improved after training (24). Richter put forward the iLead program based on the comprehensive implementation framework and all-around leadership field, which effectively improved the implementation leadership level of managers (27). This iLead program provided the foundational framework for our training program. Page et al. conducted an integrated review of the leadership education programs in the nursing field, and found that the seminar on continuous quality improvement was the most popular (28). However, this review only included 10 articles, and the participants of most included studies were formal leaders or managers. Thus, leadership training programs for informal leaders are urgently needed.

Herein, we designed a 60-class hour implementation leadership training program, including theoretical training, interactive workshops, theme reports, etc., aiming to improve the leadership skills and behaviors of nursing informal leaders and create a positive strategic organizational atmosphere, thereby supporting the effective and sustainable implementation of EBP. Meanwhile, we used Kirkpatrick’s evaluation model, which has been widely used in training sessions (29), to evaluate the effectiveness of leadership training. Additionally, we focused on the following three research objectives: (a) to evaluate informal leaders’ satisfaction with the implementation leadership training program. (b) to assess the program’s impact on evidence-based nursing beliefs and competencies. (c) to examine improvements in implementation leadership skills for evidence-based practice. Our findings may promote the successful implementation of EBP in nursing and provide a reference for the development of leadership training programs for nursing informal leaders.

## Methods

### Study design

A single-group pretest-posttest quasi-experimental design was employed, characterized by the absence of a control group. Due to pragmatic constraints (clinical scheduling conflicts), random allocation was unfeasible.

### Participants

**Figure 1:**
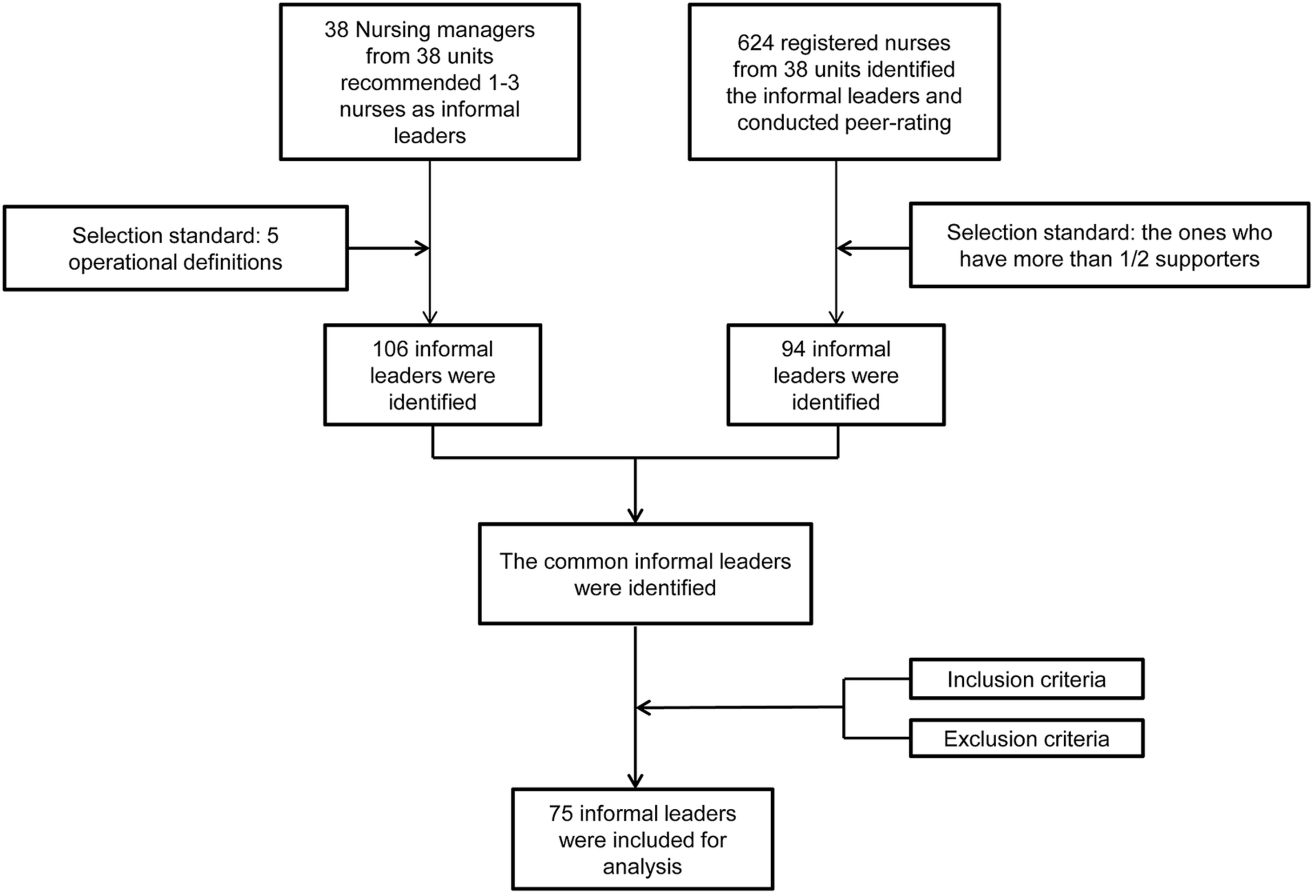
Identification of informal leaders for the implementation leadership training program.

This study was conducted at Hangzhou TCM Hospital Affiliated to Zhejiang Chinese Medical University. According to the method described by Lawson et al. (22), the identification of informal leaders was performed, which included nursing manager recommendation, peer rating, and team screening, as shown in Figure 1. *A priori power analysis* (GPower 3.1; *α* = 0.05, power = 0.8, expected Cohen’s *f* = 0.25) indicated n ≥ 68 for repeated-measures ANOVA. Our sample (*n* = 75) exceeded this threshold.

For the recommendation by nursing managers, the nursing managers of 38 nursing units recommended 1–3 informal leaders, and a total of 106 informal leaders were recommended. Based on the conceptual definition of informal leaders proposed by Downey et al. (18), we proposed the operational definition of informal leaders: (1) The academic clinical nurse educator, who shared their professional knowledge online or offline ≥ 3 times/year; (2) The main participants in nursing quality control and team development strategy; (3) Nurses who proposed patient-centered nursing measures that were often adopted and easily implemented by colleagues; (4) Nurses who hold team member together to achieve the goals of improving nursing quality, promoting nursing innovation, and improving nursing research; (5) The possible roles or qualifications of nurses included professional nurses, clinical nursing teachers (coaches), nursing supervisors, postgraduate students or champions, with an excellent qualification.

For peer rating, an electronic questionnaire survey was conducted for all registered nurses in 38 nursing units. The inclusion criteria were nurses who were directly involved in clinical nursing work and worked in the hospital for ≥ 30 days. Nurses from management posts were excluded. According to the identification methods by Lawson et al. (22) and Loughhead et al. (30), we designed a personalized online questionnaire to identify informal leaders by peer rating. The questionnaire contained an operational definition of informal leaders in peer rating and five questions. The operational definition was “Please list the names of team members (including yourself) who contribute the most to the team task.” The five questions included “My co-worker contributes to the harmony within the team and focuses on the team goals; My co-worker ensures the participation of each team member and helps to clarify their responsibilities; My co-worker helps to solve the conflicts and assists in decision making; My co-worker always offers support and is trusted by team members; My co-worker can be a spokesman for the team.” Each nurse participating in the survey could recommend and evaluate 1–3 colleagues. First, they listed the recommended candidates according to the above five questions and then scored them using Likert 5-level scoring method (totally disagree = 1 point, totally agree = 5 points). The ones who were recommended by more than half of the staff in the nursing unit were defined as informal leaders, and their average score was calculated. Nurses with an average leadership rating higher than one standard deviation above the system-wide sample mean were defined as informal leaders (22). In total, 624 nurses from 38 nursing units participated in peer rating, and 94 were identified as informal leaders after peer rating, with an average score of 21.26 ± 3.47 points.

For team screening, we screened out the common informal leaders by nursing manager recommendation and peer rating. A total of 75 nurses were finally enrolled in this study according to the inclusion and exclusion criteria. Inclusion criteria: (1) nurses who met the operational definition of informal leaders and who were identified as informal leaders by peer rating; (2) professional background: nurses with a Bachelor’s degree or above and more than 3 years of experience in clinical nursing; (3) professional consciousness: nurses who were interested in nursing EBP; (4) nurses who signed informed consent and voluntarily participated in this study. Exclusion criteria: (1) nurses with the title of manager or director; (2) nurses who participated in other research within 3 months or withdrew from the study before the end of the study.

### The training program

The training program for implementation leadership of informal leaders was designed based on the Ottawa Model of Implementation Leadership (O-MILe) (8) and behavioral leadership research and theory (31). The training program is based on the methodological frameworks of Loughead et al.’s leadership development framework (30), Huang et al.’s competency-building model (32), and Richter et al.’s evidence-based iLead framework (27), whose three core components we adopted: (1) Knowledge Acquisition (Theoretical Training): 32 h of didactic sessions on leadership principles; (2) Skill Application (Interactive Workshops): 22 h of team-based scenario simulations; (3) Behavioral Integration (Theme Report): 6 h of reflective implementation projects. This phased approach progressively links theory and practice from cognitive understanding to contextualized practice and sustained behavior change, which fully embodies the iLead framework’s core elements of experiential workshops, and reflective practice. It was conducted from April to July 2021, with a duration of 4 months and 60 class hours, including 32 class hours of theoretical training, 22 class hours of interactive workshops, and 6 class hours of theme reports (Table 1).

**Table 1:** The training program of implementation leadership for informal leaders based on O-MILe.

Model	O-MILe category	O-MILe concepts	Content	Form	Hours
Theoretical training (4 sessions per week, 16 sessions in total, 2 h each session, and 32 class hours in total)	Core knowledge	Knowledge of leadership theory	Effective communication skills; Establishing harmonious relationships; Constructive conflict management; Rational allocation of nursing human resources; Nursing safety and risk management; Service model innovation; Emotional Intelligence and Pressure Response; Leadership theory and application.	Didactic Learning	16
		Knowledge of evidence-based best practice	Evidence-based practice and core elements; Evidence resource search; Quality evaluation of literature and evidence; Theory of knowledge translation and clinical application of evidence.		8
	Core skills	Development of an implementation plan Knowledge of current practices and outcomes Knowledge of effective implementation methods Participation of clinicians, staff, and patients Setting of target goals for change	Developing an Evidence-Based Implementation plan and goals; Key aspects of the clinical application of evidence; Framework for action promotion: i-PARIHS; Case sharing of exercising leadership in promoting evidence-based practice.	Didactic Learning; Case Study Teaching	8
Interactive workshop (once a month, for a total of 22 class hours)	Relations-oriented leadership behaviors	Recognizing efforts to change Supporting change visibly and symbolically Facilitating interprofessional consensus on change Communicating with staff about clinical practice issues and EBP	Divided into 15 teams depending on the goals of quality of care change. Defining implementation projects and communication tasks based on PICO principles under the guidance of a mentor. Identifying interprofessional members who will participate in the implementation project. Assessing feedback from other colleagues and nurse leaders on the evidence-based implementation project. Training in implementation leadership behaviors to encourage staff behaviors that support implementation.	Interactive Learning; Teamwork; Workshop; Mentors	8
	Change-oriented leadership behaviors	Demonstrating commitment to change Reinforcing vision and goals of change Understanding and acting on difficulties with change Advocating for change internally and externally	Analyzing the strengths, weaknesses, and resources for project implementation based on SWOT, and identifying facilitating and hindering factors. Development of strategies to eliminate hindering factors. Implementation of institutional motivating factors for innovation. Intensive exercise: Analysis of site-specific practice gaps and redefinition of the implemented change objectives.	Interactive Learning; Teamwork Workshop; Mentors	8
	Task-oriented leadership behaviors	Clarifying roles and responsibilities Modifying documentation forms Procures resources, education, and policies to reflect the change Monitoring performances and outcomes Providing reminders Conducting regular leadership meetings	Developing a schedule for implementing the project. Developing a strategy for creating a climate for change. Discussing follow-up action plans, monitoring changes, and adjusting implementation leadership behaviors.	Interactive Learning; Teamwork Workshop; Mentors	6
Theme Report (once, with a total of 6 class hours)	Implementation of evidence-based practice	High-quality healthcare delivery	After the workshop, the 15 teams conducted a 3-month implementation project to facilitate the achievement of the change goals; The groups reported on their implementation projects and reflected on the improvement of implementation leadership in evidence-based practice.	Reflective learning; Panel presentation	6

The theoretical training was performed in April 2021 for 4 weeks (4 sessions per week, 16 sessions in total, 2 h each session, and 32 class hours in total). The core knowledge and skills of informal leaders were trained. Four graduate student supervisors with experience in evidence-based projects and two experts in the field of medical industry-university-research performed the training by using didactic learning. Each session included a brief introduction, a special lecture, and a question-and-answer session. Theoretical training cultivated three leadership competencies through evidence-based leadership frameworks: (1) Relational artistry (e.g., conflict mediation, team motivation); (2) Operational management (e.g., resource allocation, workflow optimization); (3) Self-directed leadership development (e.g., reflective practice, emotional regulation). In addition to the practical methods of evidence implementation, the training content of core skills also included the case sharing of exercising leadership to promote EBP by a nursing director.

The interactive workshop was held three times from May to July 2021, once a month, for a total of 22 class hours. The 75 informal leaders were divided into 15 teams according to their professional fields and trained for three leadership behaviors, including relations-oriented, change-oriented, and task-oriented leadership behaviors. The topic of the first seminar was relations-oriented leadership behaviors, focusing on identifying and defining the team EBP project, collecting feedback from colleagues, and determining the implementers. The topic of the second seminar was change-oriented leadership behaviors, focusing on the promotive and obstructive factors of EBP implementation, and formulating the implementation strategy. The topic of the third seminar was task-oriented leadership behaviors, which focused on the sustainability of EBP implementation. To ensure the feasibility of the project, 4 graduate student supervisors with experience in evidence-based projects and 15 ward nursing managers served as project supervisors. Under the guidance of project supervisors, the informal leaders accumulated experience in EBP implementation through group contact and team cooperation.

The theme report was performed in the last week of July 2021, with a total of 6 class hours. At this stage, 2 graduate student supervisors with experiences in evidence-based projects were invited as review experts, and 15 teams were required to report their EBP projects, including the realization of team goals and the improvement of implementation leadership. Participants maintained reflective diaries to enhance self-regulatory capacity in leadership practices, translating insights into goal-directed managerial behaviors.

### Effectiveness evaluation with Kirkpatrick’s model

Our study refers to the team’s previous research (32) and used Kirkpatrick’s Model to evaluate the training effectiveness. Two postgraduate students conducted the field survey of Kirkpatrick’s Model questionnaire at the reaction level. One nursing postgraduate student conducted the questionnaire survey at the learning level, behavior level, and result level. The investigators of our team and two graduate supervisors with experience in the evidence-based project performed the scoring of the integration system during the training.

At the reaction level, the Implementation Leadership Training Satisfaction Evaluation Questionnaire was independently designed. The questionnaire included 10 items and three dimensions, including training outline, training organization, and overall situation. The evaluation of the training outline included training goals, training effects, progressiveness and practicability of contents, training difficulty, training time design, and training methods. The evaluation of the training organization included reasonable organization arrangement, professional level of training staff, and, teaching site and facility. The degree of satisfaction was evaluated with the overall situation. Survey responses consisted of Likert scale from 1 (not at all satisfie) to 5 (completely satisfied). The training outline, training organization, and overall situation were scored 30 points, 15 points, and 5 points, respectively, with a full score of 50 points. Through two rounds of consultation, discussion, and revision by experts (3 chief nurses and 2 deputy chief nurses), the content validity index of the questionnaire was 0.93. The pilot survey was conducted on 30 informal leaders who were not included in the study. The Cronbach’s *α*coefficient was calculated as 0.885.

At the learning level, the Evidence-Based Practices Belief Scale, with Cronbach’s α coefficient of 0.895 and the content validity index of 0.629–0.870 (33), was used to evaluate evidence-based knowledge, competency, and attitude levels of informal leaders. The scale included three dimensions of basic knowledge (5 items), competency (7 items), and attitude (4 items) levels of EBP. It was developed as a 5-point Likert type scale (for 1-strongly disagree to 5-strongly agree). The total score of the scale was 80. The higher the score, the better the evidence-based nursing belief. In our study, Cronbach’s *α* coefficient of the scale was 0.872.

At the behavior level, the Evidence-Based Nursing Competence Scale was used (34), which had 4 dimensions and 23 items, including evidence retrieval and critical appraisal (7 items), evidence synthesis (5 items), evidence transfer (3 items), and, situational evaluation and evidence application (8 items). The Cronbach’s *α* coefficient of the scale was 0.951, and of each dimension was 0.855–0.916. The scale uses a Likert-4 scale, ranging from 1 (strongly disagree) to 4 (strongly agree), with a maximum score of 92 points. The higher the score, the stronger the evidence-based nursing ability. In our study, Cronbach’s α coefficient was 0.912.

At the result level, the Implementation Leadership Scale with Cronbach’s α coefficient of 0.86 ~ 0.95 was adopted (35). This scale comprises 12 items assessing informal leaders’ implementation leadership in evidence-based practice across four domains: Proactive Leadership (3 items), Knowledgeable Leadership (3 items), Supportive Leadership (3 items), and Perseverant Leadership (3 items). Responses are rated on a 5-point Likert scale (0–4), ranging from “strongly disagree” to “strongly agree,” yielding a total score range of 0–48. Higher scores indicate stronger implementation leadership competencies among nursing informal leaders. In our study, Cronbach’s *α* coefficient was 0.924. Additionally, the process evaluation of the training was carried out through the integration system, with a full score of 50 points. The scores for attendance, participation in course interaction, and special topic sharing were 10 points each, and the score for reflective diaries was 20 points. After the training, the written debriefing reports and theme reports of the EBP project were submitted and evaluated, with a full score of 50 points. The final score was the combination of the process evaluation score and the debriefing score, with a full score of 100 points.

### Program implementation costs

The 60-h training program incurred three primary resource investments: (1) Personnel costs: Since all the trainees were willing to sacrifice personal time for training, the theoretical training in this study was arranged for 2 h each time after work. The program comprised 28 paid work hours (theoretical training and theme report) and 32 off-duty hours (theoretical training). Unit coverage cost was CNY 63,000 (CNY 30/h × 28 h × 75 nurses), calculated via hospital payroll data. (2) Instructional resources: External expert honoraria was CNY 35,000 (20% discount for institutional partnership). (3) Indirect costs: Utilized the existing hospital infrastructure to provide teaching equipment (such as projectors, simulation toolkits), printed materials (manuals, assessment tools), and access to e-learning platforms, etc.

### Data collection

The data of scale questionnaires at the reaction, learning, behavior, and result levels were collected by two master’s students at the pre-training and at the end of the post-training. Pre-training surveys were administered in-person 1 week before program initiation during unit briefings. Post-training surveys were completed immediately after the final session. The baseline characteristics of the study participants were collected, including sex, age, highest degree, professional title, years of working, and, nursing responsibilities and departments. Survey dissemination occurred via the online platform WJX.^1^ Questionnaire links and informed consent documents were distributed through WeChat groups, enabling participants to independently complete surveys using electronic devices. All questionnaires employed unique participant codes to ensure anonymity. Researchers had no access to identifiable information, preventing linkage between responses and individual identities.

The questionnaire design mandated responses for every item, with automated reminders triggered for missing answers. Upon completion of data collection, two research team members scrutinized the acquired datasets. Data entry and subsequent analysis were performed using standardized statistical software packages.

### Statistical analysis

SPSS 23.0 statistical software was used for data analysis. The mean ± standard deviation, frequency, and percentage were used to describe the scores of the integration system, training satisfaction, Evidence-Based Practices Believe Scale, Implementation Leadership Scale, and Evidence-Based Numbering Competence Scale. The paired t-test or paired Wilcoxon signed-rank test was used to analyze the changes in scores at the reaction level, learning level, behavior level, and result level before and after training. The degrees of freedom were calculated by the formula of (independent values—statistical values). *p* < 0.05 indicates a statistically significant difference.

## Results

### Participant characteristics

The characteristics of the informal leaders included in our study are described in Table 2. Most participants were female (96%), with mean age (35.8 ± 4.5) years. No informal leaders withdrew from the study.

**Table 2:** Demographic characteristics of the informal leaders (*n* = 75).

Characteristic	N	%
Gender		
Female	72	96.00
Male	3	4.00
Age		
25–30 years	10	13.33
31–40 years	52	69.33
41–50 years	13	17.34
Working years		
3–10 years	26	34.67
11–20 years	44	58.67
≥21 years	5	6.66
Highest degree		
Bachelor’s degree	61	81.33
Master’s degree	14	18.67
Professional title		
Senior clinical rank	63	84.00
Mid-level clinical rank	12	16.00
Position		
Nurse educator	13	17.33
Care facilitator	13	17.33
Bedside nurse	49	65.34
Department		
Internal medicine	29	38.67
General surgery	22	29.33
Gynecology and pediatrics	8	10.67
Critical care unit	6	8.00
Operating rooms, outpatient clinics, and supply rooms	6	8.00
Others	4	5.33

### Effect of implementation leadership training at the reaction level

The Implementation Leadership Training Satisfaction Evaluation Questionnaire was used to evaluate the effectiveness of training at the reaction level. The results showed that the satisfaction of the study participants scored 48.42 ± 2.38 points (total score of 50 points). The score for the training outline was 29.12 ± 1.53 points (full score of 30 points), for training organization was 14.47 ± 0.95 points (full score of 15 points), and for overall situation was 4.84 ± 0.37 points (full score of 5 points).

### Effect of implementation leadership training at the learning level

At the learning level, the Evidence-Based Practices Belief Scale scores of the study participants increased from (59.91 ± 5.96) points before training to (69.24 ± 5.32) points after training. The difference between them was statistically significant (*t* = 62.274, *p* < 0.001; Table 3).

**Table 3:** Evidence-based practices belief scale scores before and after training (*n* = 75).

Item	Pre-training, mean ± SD	Post-training, mean ± SD	Difference (95% CI)	*Cohen’s d*	*t* value	*p* value
Evidence-based practice basic knowledge level	18.20 ± 2.61	21.33 ± 2.37	3.13 [2.99,3.27]	1.25	45.203	0.000^*^
Evidence-based practice competency level	27.44 ± 3.32	31.53 ± 2.88	4.09 [3.87,4.31]	1.32	36.886	0.000^*^
Evidence-based practice attitude level	14.27 ± 2.64	16.37 ± 2.46	2.11 [1.97,2.25]	0.83	30.122	0.000^*^
Total scale^a^	59.91 ± 5.96	69.24 ± 5.32	9.33 [9.01,9.63]	1.65	62.274	0.000^*^

SD, standard deviations. *p < 0.001.

a Scale range = 16–80.

### Effect of implementation leadership training at the behavior level

At the behavior level, the scores of the Evidence-Based Nursing Competence Scale score increased from (56.37 ± 7.15) points before training to (74.47 ± 5.75) points after training, with a statistically significant difference (*t* = 55.290, *p* < 0.001; Table 4).

**Table 4:** Evidence-Based Nursing Competence Scale scores before and after training (*n* = 75).

Item	Pre-training, mean ± SD	Post-training, mean ± SD	Difference (95% CI)	*Cohen’s d*	*t* value	*p* value
Evidence retrieval and critical appraisal	17.65 ± 2.98	21.81 ± 2.47	4.16 [3.66,4.65]	1.52	16.659	0.000*
Evidence synthesis	9.95 ± 2.85	16.51 ± 2.55	6.56 [6.28,6.83]	2.43	47.812	0.000*
Evidence transfer	7.15 ± 2.10	10.23 ± 1.92	3.08 [2.961,3.24]	1.53	37.460	0.000*
Situational assessment and evidence implementation	21.63 ± 3.68	25.92 ± 3.36	4.29 [4.13,4.45]	1.22	53.647	0.000*
Total scale^a^	56.37 ± 7.15	74.47 ± 5.75	18.09 [17.44,18.75]	2.79	55.290	0.000*

SD, Standard deviations. *p < 0.001.

a Scale range = 23–92.

### Effect of implementation leadership training at the result level

At the result level, the Implementation Leadership Scale score of the study participants was (30.01 ± 3.24) points before training, which significantly increased to (38.88 ± 2.76) points after training (*t* = 51.337, *p* < 0.001; Table 5).

**Table 5:** Implementation leadership scale scores before and after training (*n* = 75).

Item	Pre-training, mean ± SD	Post-training, mean ± SD	Difference (95% CI)	*Cohen’s d*	*t* value	*p* value
Proactive Leadership	7.05 ± 1.68	9.36 ± 1.32	2.31 (2.11, 2.51)	1.53	23.030	0.000^*^
Knowledgeable Leadership	7.62 ± 1.59	9.84 ± 1.47	2.22 (2.09, 2.35)	1.45	33.080	0.000^*^
Supportive Leadership	7.98 ± 1.26	9.99 ± 1.17	2.01 (1.91, 2.11)	1.65	40.429	0.000^*^
Perseverant Leadership	7.38 ± 1.71	9.57 ± 1.53	2.19 (0.24, 2.34)	1.35	28.885	0.000^*^
Total scale^a^	30.01 ± 3.24	38.88 ± 2.76	8.87 (8.53, 9.21)	2.95	51.337	0.000^*^

SD, Standard deviations. *p < 0.001.

a Scale range = 0–48.

The total score of the post-training integration system assessment was (79.65 ± 5.15) points, of which the process assessment score was (40.61 ± 3.57) points and the debriefing score was (39.04 ± 3.94) points. Totally, 75 participants scored above 70 points, and 38 scored 80–89 points, indicating a good overall assessment.

## Discussion

Excellent nursing leadership can narrow the gap between nursing theory and practice as well as promote EBP and organizational change and development (36). It is recommended that nurses at all levels should participate in leadership training and decision-making, and put forward suggestions to improve the quality of nursing (37). However, most of the studies (38–40) on nursing leadership training are for nursing managers with administrative positions. One study reported undergraduate nurse education (41). However, there are few studies on the training of nursing leadership of clinical nurses (28). The innovation of this study is that the nursing informal leaders were identified through manager recommendation, peer rating, and team screening, and the implementation leadership training program was developed. The training program included the contents of nursing, evidence-based medicine, and management. The training was carried out via theoretical training, interactive workshops, and theme reports. Meanwhile, the EBP project ran through the training, which promoted the training of implementation leadership. In addition, Kirkpatrick’s evaluation model (29) was used for the evaluation of training effectiveness. Our findings may provide a scientific basis for training and improving the leadership of informal leaders.

The results of this study showed that the overall satisfaction of informal leaders with the implementation leadership training program was at a high level, and no one withdrew from the study, indicating that the responses of the participants to the training are positive. This is consistent with the results of Proctor et al. (24), implying that a four-month program is acceptable and applicable for leadership training. Effective nursing leadership training necessitates integrating theoretical frameworks with implementation leadership praxis. In this study, EBP projects were conducted in small groups following theoretical instruction on core implementation leadership competencies. This training approach, which is grounded in implementation leadership theory (8), fosters transformational leadership behaviors that enable informal leaders to support evidence-based change. By integrating the core change elements of providing feedback and encouragement, demonstrating commitment, and reinforcing the vision and willingness to change into the training design (8, 25), this study creates an organizational climate conducive to the implementation of EBP, which in turn promotes informal leaders engagement and satisfaction.

Then, we found that the scores of the evidence-based nursing belief and evidence-based nursing ability of informal leaders after training were higher than those before training, indicating that the training program can not only improve the belief of participants in EBP, including knowledge, ability, and attitude but also improve their evidence-based nursing ability. Similar results have also been reported by previous studies. For example, Varnell et al. found that an 8-week education program improved the attitude of nurses in critical care units toward EBP (42). Through a randomized controlled trial, D’Souza et al. confirmed that EBP training was effective in improving the knowledge, attitude, and ability of EBP of nursing educators (43). Proctor et al. verified the positive effect of the TRIPLE project in improving the knowledge and behavior of participants in implementing EBP; and, they found that task-oriented leadership was more effective in affecting the implementation of health interventions (22). In this study, the interactive workshop was designed for the three types of behaviors required for the implementation of EBP proposed in the O-MILe model. The project research, design, analysis, implementation, and report writing were conducted through specific evidence-based projects, fully mobilizing the practical enthusiasm of each participant and improving the ability of informal leaders in recognizing and analyzing problems during implementing evidence-based projects. After training, the participants may put forward unique opinions, have enhanced awareness of their advantages, job responsibilities, decision-making, hospital development, and job objectives, and mobilize the resources of their own, the team, and the supporting platform, thus achieving the clinical implementation of EBP project in an organized and planned way.

O-MILe is a theoretical model for developing implementation leadership, and Implementation Leadership Scale is an empirical verification tool for evaluating implementation leadership (8). Our results showed that the Implementation Leadership Scale score of informal leaders after training was higher than that before training, and the overall performance evaluated by the integration system was good, which confirms the scientific value of this training program in improving implementation leadership. Our findings demonstrate that implementation leadership training effectively enhances EBP capabilities among informal nurse leaders—extending Richter et al.’s (27) managerial intervention framework to frontline nursing contexts. Whereas Richter focused on formal healthcare managers, our study adapted their multidimensional leadership development approach (combining didactic, workshop, and reflective elements) to empower informal leaders through peer-driven learning networks. This training approach highlights the unique value of leadership interventions that target the relational influence and clinical embeddedness of informal nurse leaders compared to standard training models. Kim et al. proposed “making their voices heard” as a means to improve nursing leadership education (44). In the present study, we set up group activities at the interactive workshop and theme-sharing stages to promote participants to share their ideas with colleagues, seniors, descendants, and people from other disciplines, while strengthening their leadership. Meanwhile, various methods of cooperative experience-driven education (44) were used in the training program of this study, including support and encouragement from leaders, tutorial teaching, team-based cooperative training, etc., thus improving the external conditions of EBP in nursing. Furthermore, reflection and interaction can promote personal ability development and enhance self-confidence (45). In the process evaluation, this study initiated and promoted the dialog, discussion, and reflection of informal leaders through “reflective diary” and “interactive workshop and theme sharing,” which helps informal leaders learn to experience and exert influence. This study enhanced the implementation leadership of informal leaders via the training program, which may be conducive to the successful implementation of EBP within the organization.

Informal nurse leaders are indispensable catalysts for improving the quality of patient care and job satisfaction (21), a role that is functionally distinct from formal leaders, who derive their power from positional authority. Informal leaders operate on the front lines, identifying context-specific barriers that are invisible to formal management, and acting as purveyors of evidence to carry upward voices to formal leaders (46). Although implementing leadership training requires a significant investment, it accelerates EBP integration into clinical workflows, enhances care quality, resolves complex problems, and reduces adverse events—collectively advancing patient safety (47). While the evidence regarding the return on investment (ROI) of EBP is still emerging (48), recent studies have shown positive prospects (49). Therefore, healthcare administrators should support the development of informal leaders to systematically build capacity for EBP, thereby promoting patient safety and enhancing reputation.

This study has some limitations. First, despite anonymous data collection, cultural hierarchies in healthcare settings may inflate self-reported gains. Future evaluations should include objective metrics to complement self-assessment. Second, the absence of longitudinal follow-up precludes assessment of sustained organizational impact. Consequently, the durability of leadership behavioral changes and their translation into lasting EBP implementation remains unverified. Third, the absence of qualitative analysis limits deeper understanding of how behavioral changes occurred. For example, critical incident technique analysis could reveal why EBP implementation training showed the great improvement, semi-structured interviews could explore the implementation obstacles encountered by informal leaders in EBP projects. Fourth, the single-group pretest-posttest design restricts causal inference. Observed improvements may reflect concurrent organizational initiatives, or Hawthorne effects. In the future, multi-center, large-sample empirical studies with control groups will be needed.

## Conclusion

Our findings demonstrate that the implementation leadership training program is highly accepted by informal leaders and can effectively improve the evidence-based knowledge and ability of implementation leadership. The training program meets the leadership training needs of informal leaders and provides the localization method for identifying informal leaders as well as the framework and evidence for the suitability and effectiveness of training intervention strategies, laying a foundation for the development and evaluation methods of implementation leadership training in the future. In the future, this training program may be applied to nursing EBP projects on a large scale, which will have a more direct and greater impact on practice change and ensure that nurses play an influential role in health policy formulation and decision-making.

## Data Availability

The raw data supporting the conclusions of this article will be made available by the authors, without undue reservation.

## Footnotes

^1^ https://www.wjx.cn
